# Molecular Identification and Genotyping of Phytoplasmas Infecting Medicinal and Aromatic Plants in Northern Italy

**DOI:** 10.3390/microorganisms13071444

**Published:** 2025-06-21

**Authors:** Camilla Barbieri, Abdelhameed Moussa, Alessandro Passera, Paola Casati, Piero Attilio Bianco, Fabio Quaglino

**Affiliations:** Department of Agricultural and Environmental Sciences–Production, Landscape, Agroenergy, University of Milan, Via Celoria 2, 20133 Milan, Italy; camilla.barbieri@unimi.it (C.B.); abdelhameed.moussa@unimi.it (A.M.); alessandro.passera@unimi.it (A.P.); paola.casati@unimi.it (P.C.); piero.bianco@unimi.it (P.A.B.)

**Keywords:** MAPs, Mollicutes, 16SrXII-A, multilocus sequence typing, recombination

## Abstract

During field surveys carried out in 2021 at two farms in Lombardy (North Italy), leaf samples were collected from 113 plants (both symptomatic and asymptomatic) belonging to 18 medicinal and aromatic species. Amplification and nucleotide sequence analyses of the 16S rRNA gene revealed the presence of ‘*Candidatus* Phytoplasma solani’ (subgroup 16SrXII-A) in 69 plants (61% infection rate) belonging to 14 of the 18 examined species. Among the 14 infected species, only *Nepeta cataria* L. exhibited symptoms including leaf and stem reddening. Molecular typing analyses showed that ‘*Ca*. P. solani’ strains identified in this study constitute a genetically homogeneous population, carrying the *stamp* gene sequence variant St5 and the new *vmp1* gene sequence variant Vm93. Phylogenetic analyses showed that ‘*Ca*. P. solani’ strain St5/Vm93 belongs to the cluster b-II, associated with the bindweed-related pathosystem. In silico-translated Vmp1 protein sequence alignment suggested that ‘*Ca*. P. solani’ strain St5/Vm93 could be generated by recombination events between ‘*Ca*. P. solani’ strains co-infecting the same host. The results suggested future research investigating the diffusion and the ecology of ‘*Ca*. P. solani’ strain St5/Vm93 in agroecosystems (including other crops), and its effect on the composition of biologically active compounds in aromatic and medicinal plants.

## 1. Introduction

The category of medicinal and aromatic plants (MAPs) includes “botanicals that provide people with medicines to prevent disease, maintain health or cure aliments” (definition by FAO’s Medicinal and Aromatic Plant Working Group), playing important economic, socio-cultural, and ecological roles worldwide [1]. Even if medicinal and aromatic plants are often referred to as synonymous, it can be more appropriate to state that medicinal plants carry components beneficial to health (biologically active compounds), while aromatic plants produce essential oils mainly utilized in sectors such as perfume, cosmetics, toothpaste, aromatherapy, soap, beverage, and food industries [2,3]. Currently, researchers are looking for MAP active ingredients for weed and disease control in agriculture [4,5,6] or for use as natural colorants in industries [7]. Considering the current globally increasing demand for MAPs and their derived products, biotic stresses constitute important limiting factors for yield quantity and biologically active compound quality. In detail, MAP diseases associated with phytoplasmas represent a major threat that can severely damage yield and plant longevity and alter the composition of biologically active compounds [8,9].

Phytoplasmas are cell wall-less bacteria, belonging to the Mollicutes class, living as obligate pathogens within the phloem sieve tube elements of plants [10]. They can be transmitted from plant to plant by phloem-feeding insects through persistent propagative modality [11]. Due to the difficulty of cultivation in axenic conditions [12], provisional phytoplasma taxonomy is defined by guidelines based on nucleotide sequence analyses of the 16S rRNA gene and other housekeeping genes (*tufB*, *rplV*-*rpsC*, *secY*, *secA*, *groEL*), and/or average nucleotide identity (ANI) calculated on complete/draft phytoplasma genomes (if available) [13,14]. Up to now, more than 50 species have been described within the ‘*Candidatus* Phytoplasma’ genus [13,15,16]. A parallel phytoplasma classification scheme (16Sr group/subgroup classification) is based on similarity coefficients obtained by comparison of restriction fragment length polymorphism (RFLP) collective patterns of 16S rRNA gene amplicons [17,18]. Up to now, phytoplasma-associated diseases, causing yellowing/reddening, witches’ broom, and decline, have been reported mostly in Europe and Southeast Asia in more than 70 MAP species, belonging principally to Apiaceae and Asteraceae [8,19]. The main phytoplasmas associated with MAP diseases are ‘*Ca*. Phytoplasma asteris’ (subgroup 16SrI-B), reported in North America, Europe, and Asia; ‘*Ca*. Phytoplasma solani’ (subgroup 16SrXII-A), reported in Europe; ‘*Ca*. Phytoplasma citri/aurantifolia’ (subgroup 16SrII-B); and ‘*Ca*. Phytoplasma australasiaticum/australasia’ (subgroup 16SrII-D), reported in Asia and Australia [8,19].

The aim of this study was to identify and type by molecular analyses the phytoplasmas infecting MAP species in the Lombardy region, northern Italy.

## 2. Materials and Methods

### 2.1. Field Surveys and Plant Sample Collection

Surveys for phytoplasma-like symptoms were carried out in July 2021 at two farms (named Farm-1 and Farm-2) in the Lombardy region (northern Italy). Farm-1 is a biodynamic farm in La Valletta Brianza, Lecco province (45.72226, 9.370752000000039), spanning over 20 hectares of terraced hills, which include forests, meadows, vineyards, orchards, and gardens. Numerous species of MAPs are cultivated or grow spontaneously within this highly complex agroecosystem. Farm-2 is an organic farm in Solto Collina, Bergamo province (45.781785, 10.025203), specializing in the production of donkey milk for cosmetic purposes, and includes vegetable gardens and orchards where numerous MAP species are cultivated. During the inspections, stem, leaf, and/or flower samples were collected from symptomatic and symptomless plants representing the diversity of the MAP species present at Farm-1 and Farm-2. Collected plant samples were stored at 4 °C for transport to the laboratories of the Department of Agricultural and Environmental Sciences (DiSAA), University of Milan, and stored at −30 °C until further analyses.

### 2.2. Phytoplasma Identification

Total nucleic acids (TNAs) were extracted from 1 g of stem, leaf, and/or flower tissue collected from each of the MAP samples using the CTAB-based method published by Angelini and colleagues [20]. The quantity and quality of extracted TNAs were measured using a NanoDrop ND-1000 Spectrophotometer (Thermo Fisher Scientific, Monza, Italy). TNAs (50 to 100 ng) were employed as templates in nested polymerase chain reaction (PCR)-based amplification of phytoplasma 16S rRNA gene using primer pair P1/P7 (direct PCR) [21,22], followed by primer pair R16F1/R16R0 (nested PCR) [23,24]. Reaction mixtures were carried out utilizing GoTaq^®^ DNA Polymerase (Promega Italia, Milan, Italy), and amplification profile conditions were as previously described [21,22,23,24]. TNA extracted from healthy periwinkle [*Catharanthus roseus* L. (G. Don)] and a reaction mixture devoid of TNA were utilized as negative controls. No positive controls (TNA from phytoplasma-infected periwinkles) were employed to avoid reaction contamination. Nested PCR products were electrophoresed on 1% agarose gel in Tris-borate–EDTA (TBE) buffer stained with Gel-Red (Biotium, Fremont, CA, USA) and visualized using a UV transilluminator (Gel Doc EZ Imager, Bio-Rad, Hercules, CA, USA).

All obtained R16F1/R16R0 amplicons were sequenced in both directions by a commercial sequencing service (Eurofins Genomics, Ebersberg, Germany). Nucleotide sequences of each amplicon were assembled using the “Contig Assembling Program” function, trimmed to the annealing sites of the primers R16F1/R16R0, and aligned to obtain a consensus sequence in the software BioEdit, version 7.1.3.0 [25]. To attribute MAP-infecting phytoplasmas to ‘*Candidatus* Phytoplasma’ species, consensus sequences were aligned with previously described reference strains of ‘*Candidatus* Phytoplasma’ species (16S rRNA gene Accession Numbers available in Figure 2) by ClustalW Multiple Alignment, and a sequence identity matrix was generated in the software BioEdit. Additionally, the obtained alignment was utilized for phylogenetic analyses by the maximum likelihood method and Tamura–Nei model, bootstrap replicated 1000 times, in the software MEGA X version 10.2.6 [26]. To establish the 16Sr group/subgroup affiliation, in silico restriction fragment length polymorphism (RFLP) analysis was conducted on 16S rRNA gene consensus sequences using the *i*PhyClassifier tool (https://plantpathology.ba.ars.usda.gov; accessed on 22 September 2022) [17,18] and confirmed by in vitro digestion of R16F1/R16R0 amplicons using the enzyme *Mse*I (New England Biolabs, Ipswich, MA, USA), following manufacturer’s instructions. Digestion fragments were electrophoresed on 3% agarose gel in TBE buffer stained with Gel-Red and visualized by the UV transilluminator.

### 2.3. Molecular Typing of Identified Phytoplasmas

‘*Candidatus* Phytoplasma solani’ (CaPsol) strains identified in MAPs were typed by nucleotide sequence analyses of *stamp* and *vmp1* genes, and amino acid sequence analyses of in silico-translated Stamp and Vmp1 proteins. TNAs extracted from phytoplasma-infected MAPs were utilized in nested PCRs for amplifying (i) *stamp* gene, using primer pairs StampF/StampR0 followed by StampF1/StampR1 [27], and (ii) *vmp1* gene, using primer pairs StolH10F1/StolH10R1 [28] followed by TYPH10F/TYPH10R [29]. Reaction mixtures were carried out utilizing GoTaq^®^ DNA Polymerase (Promega Italia, Milan, Italy), and amplification profile conditions were as previously described [27,28,29]. PCR controls and amplicon visualization were as described above for the 16S rRNA gene. To attribute CaPsol strains to *vmp1* RFLP types (V types), TYPH10F/TYPH10R amplicons were digested with the enzyme *Rsa*I [28,29] in accordance with the manufacturer’s instructions (New England Biolabs). Digestion fragments were electrophoresed on 3% agarose gel in TBE buffer stained with Gel-Red and visualized by the UV transilluminator. Attribution to V types was determined by comparison of *Rsa*I-RFLP profiles with *vmp1* gene digestion profiles previously described and in accordance with SEE-ERANET nomenclature [28,29].

All StampF1/StampR1 (*stamp* gene) and TYPH10F/TYPH10R (*vmp1* gene) amplicons were sequenced in both directions by a commercial sequencing service (Eurofins Genomics). Nucleotide sequences of each amplicon were assembled using the Contig Assembling Program, trimmed to the annealing sites of the primers StampF1/StampR1 (*stamp* gene) and TYPH10F/TYPH10R (*vmp1* gene), and aligned to obtain a consensus sequence in the software BioEdit [25]. *Stamp* and *vmp1* gene consensus sequences were aligned with previously reported *stamp* and *vmp1* gene sequence variants (St1 to St70 for *stamp* gene; Vm1 to Vm92 for *vmp1* gene) [30,31,32,33] using ClustalW Multiple Alignment and analyzed by Sequence Identity Matrix in the software BioEdit. As in previous studies [30,31,32,33], sequences belonging to the same variant share 100% nucleotide sequence identity. Moreover, to confirm the attribution to V types by in vitro RFLP analysis (see above), TYPH10F/TYPH10R nucleotide sequences were digested in silico with the enzyme *Rsa*I in the software pDRAW32 (http://acaclone.com/; accessed on 22 September 2022) and compared with virtual *Rsa*I profiles of V types previously reported [28,30].

Nucleotide sequences of *stamp* and *vmp1* genes, representative of phytoplasmas identified in this study and previously reported in literature [30,31,32,33], were utilized for phylogenetic analyses by the minimum evolution method and Jukes–Cantor model, bootstrap replicated 1000 times, in the software MEGA X [26].

Moreover, *stamp* and *vmp1* gene sequence variants, identified in MAP-infecting phytoplasma strains, were translated in silico in the software BioEdit and searched for the presence of synonymous and nonsynonymous single nucleotide polymorphisms (SNPs) or other modifications (insertions or deletions).

## 3. Results and Discussion

### 3.1. Phytoplasma-like Symptoms Observed During Field Surveys

**Figure 1:**
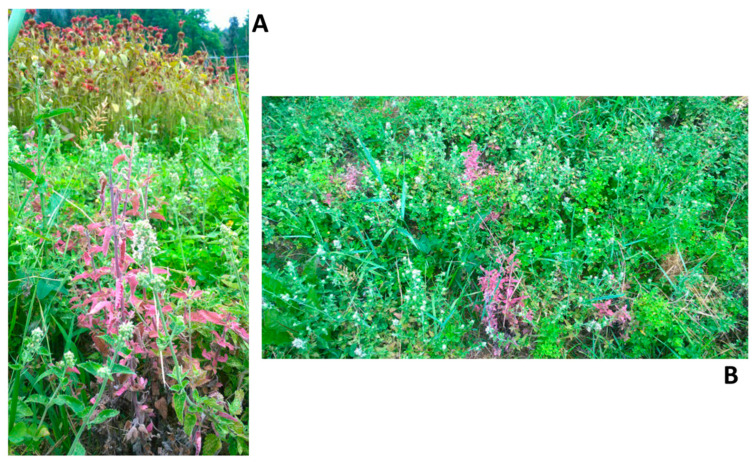
Symptoms observed in *Nepeta cataria* L. (**A**) and distribution of symptomatic plants (**B**).

During the field surveys, stem, leaf, and/or flower samples were collected from 60 plants belonging to 14 MAP species at Farm-1 and from 53 plants belonging to 9 MAP species at Farm-2 (Table 1), for an overall total of 113 plants of 18 MAP species (5 species were present at both farms: *Hypericum perforatum* L., *Lavandula* sp. L., *Melissa officinalis* L., *Mentha* sp. L., *Urtica dioica* L.). Only 7 plants of the species *Nepeta cataria* L. out of 113 collected plants of 18 MAP species showed phytoplasma-like symptoms such as leaf and stem reddening (Figure 1). All the other 106 samples were collected from symptomless plants.

### 3.2. Identification of ‘Ca. Phytoplasma solani’ in Aromatic and Medicinal Plants

Nested PCR-based amplification of the 16S rRNA gene revealed the presence of phytoplasma infection in 69 out of 113 examined plants (infection rate 61%), belonging to 14 out of 18 considered MAP species. In detail, 40 plants out of 60 (infection rate 67%) and 29 plants out of 53 (infection rate 55%) were found to be phytoplasma-infected at Farm-1 and Farm-2, respectively. Considering *N. cataria*, only the 7 plants exhibiting leaf and stem reddening were found positive to phytoplasma 16S rRNA gene amplification (infection rate 47%). Infection rate of the remnant 13 phytoplasma-infected MAP species, which did not show any symptoms, ranged from 8% (*Urtica dioica* L.) to 100% (*Artemisia absinthium* L., *H. perforatum*, *Matricaria recutita* L., *M. officinalis*, *Rosmarinus officinalis* L., *Salvia officinalis* L., *Levisticum officinale* W.D.J. Kock). No amplifications were observed in all the plant samples examined of the species *Achillea millefolium* L. (one sample), *Calendula officinalis* L. (five samples), *Echinacea purpurea* (L.) Moench (five samples), and *Inula salicina* L. (five samples) (Table 1). Robustness of PCR reaction results was proved by the absence of amplification in the negative controls.

**Figure 2:**
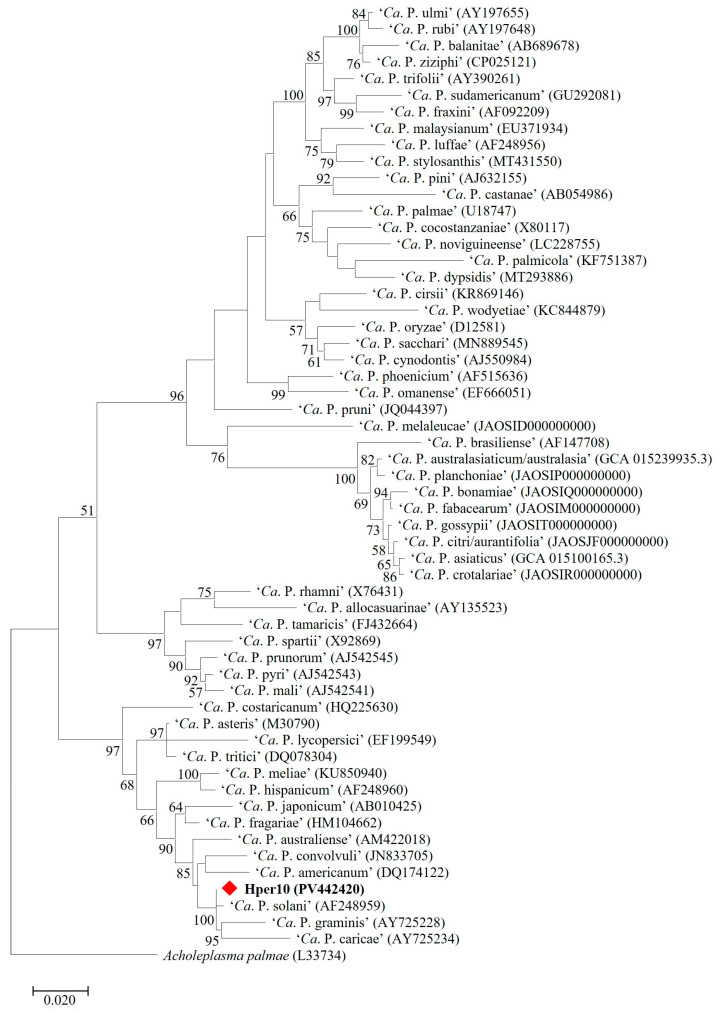
Phylogenetic tree of 16S rRNA gene nucleotide sequences inferred using the maximum likelihood method and Tamura–Nei model. The tree with the highest log likelihood (−12,065.20) is shown. Initial tree(s) for the heuristic search were obtained automatically by applying the neighbor-join and BioNJ algorithms to a matrix of pairwise distances estimated using the maximum composite likelihood (MCL) approach and then selecting the topology with superior log likelihood value. The tree is drawn to scale, with branch lengths measured in the number of substitutions per site. This analysis involved 58 nucleotide sequences. There were a total of 1427 positions in the final dataset. The Hper10 (PV4424209) strain, written in bold, represents the MAP-infecting ‘*Ca*. P. solani’ strains.

**Figure 3:**
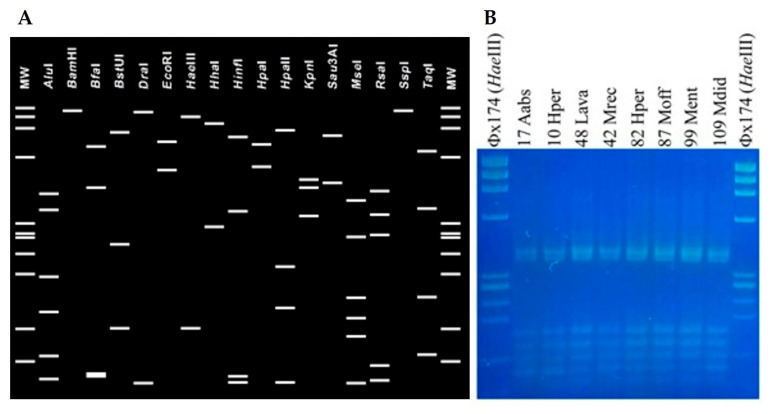
Collective RFLP profile obtained by in silico digestion of 16S rRNA sequence (PV442420) of ‘*Ca*. P. solani’ strains identified in MAPs (**A**), and *Mse*I-RFLP profiles in 3% agarose gel obtained by digestion of 16S rRNA gene amplicons of ‘*Ca*. P. solani’ strains identified in MAPs (**B**).

Nucleotide sequence analyses of R16F1/R16R0 amplicons showed that phytoplasma strains identified in 69 MAPs shared an identical 16S rRNA gene sequence (GenBank Accession Number PV442420). Alignment with 16S rRNA gene sequences of ‘*Ca*. Phytoplasma’ reference strains and sequence identity calculation revealed that MAP-infecting phytoplasma strains belong to the species ‘*Ca*. Phytoplasma solani’, showing the highest sequence identity value (99.6%) in comparison with the reference strain STOL (GenBank Accession Number AF248959). In detail, 16S rDNA nucleotide sequence of ‘*Ca*. P. solani’ strains identified in MAPs are distinct from the reference strain STOL by four single nucleotide polymorphisms (SNPs) at positions 504 (T/A), 595 (A/G), 888 (C/T), and 1084 (T/C) from the annealing site of the primer R16F1. Phylogenetic analyses reinforced the species attribution (Figure 2). As expected, in vitro and in silico RFLP analyses allowed classifying ‘*Ca*. P. solani’ strains, identified in the present study, within the taxonomic subgroup 16SrXII-A (Figure 3) [34].

Among the 14 phytoplasma-infected MAP species (Table 1), *Filipendula ulmaria* (L.) Maxim. and *Nepeta cataria* L. were found for the first time in the present study as plant hosts of ‘*Ca*. P. solani’; the other 12 MAP species (*Artemisia absinthium* L. [35], *Hypericum perforatum* L. [36], *Lavandula* sp. L. [37], *Levisticum officinale* W.D.J.Koch [38], *Matricaria recutita* L. [38], *Melissa officinalis* L. [38], *Mentha* sp. L. [39], *Monarda didyma* L. [40], *Rosmarinus officinalis* L. [41], *Salvia officinalis* L. [42], *Urtica dioica* L. [43], *Valeriana officinalis* L. [44]) were already reported as ‘*Ca*. P. solani’ hosts in Europe. Among the four MAP species found not infected by phytoplasmas (Table 1), *Achillea millefolium* L., *Calendula officinalis* L., and *Echinacea purpurea* (L.) Moench were found as ‘*Ca*. P. solani’ hosts in previous studies [33,38,45], while *Inula salicina* L. was never found infected by phytoplasmas [8,9].

‘*Ca*. P. solani’, one of the main phytoplasmas largely reported in association with MAP diseases [8], is also associated with important diseases of crops in Europe (i.e., grapevine bois noir, corn reddening, lavender decline, stolbur), and is plant-to-plant transmitted mainly by the insect vector *Hyalesthes obsoletus* Signoret [43,46].

### 3.3. Molecular Typing Revealed a New ‘Ca. Phytoplasma solani’ Genotype Infecting MAPs

Nested PCRs allowed for amplifying StampF1/StampR1 and TYPH10F/TYPH10R fragments from all 69 MAPs found infected by ‘*Ca*. P. solani’. No amplification was obtained for negative controls.

**Figure 4:**
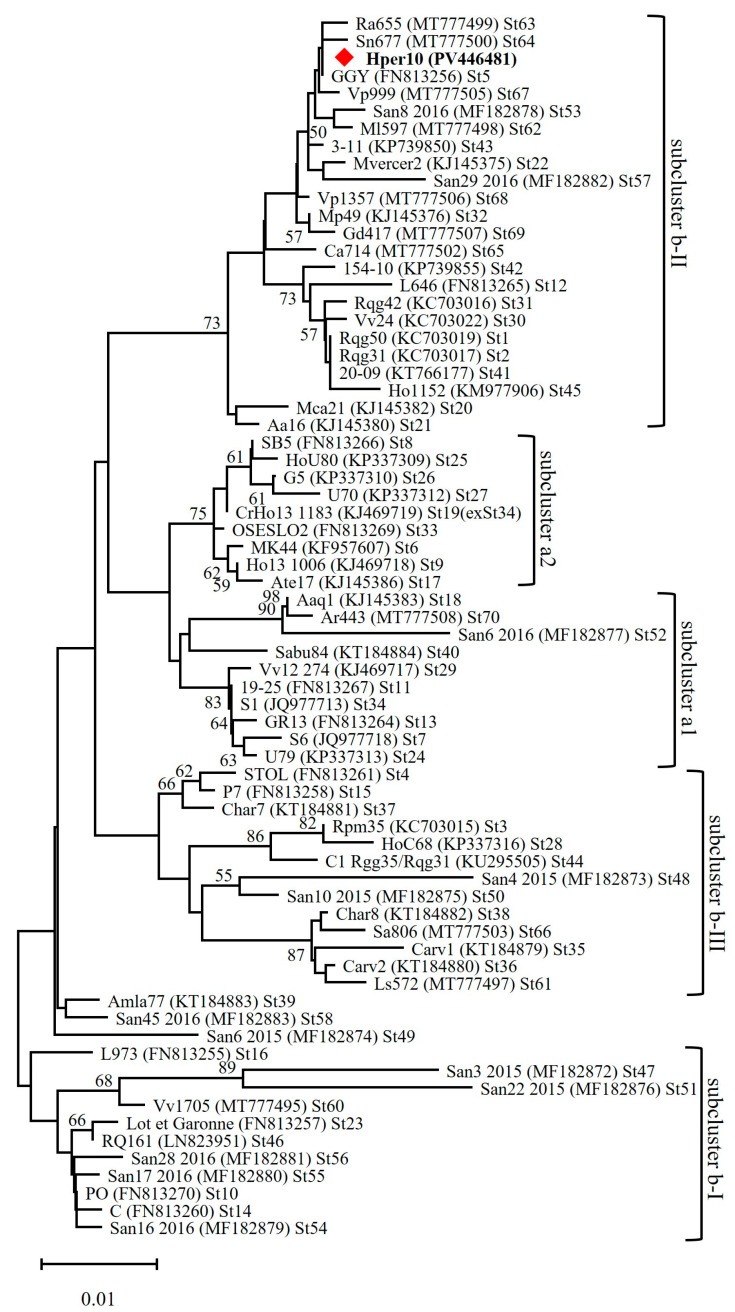
Phylogenetic tree of *stamp* gene nucleotide sequences inferred using the minimum evolution method with 1000 bootstrap replicates. The evolutionary distances were computed using the Jukes–Cantor method. The ME tree was searched using the close-neighbor-interchange (CNI) algorithm at a search level of 1. The neighbor-joining algorithm was used to generate the initial tree. This analysis involved 70 nucleotide sequences. There were a total of 495 positions in the final dataset. Hper10 (PV446481), written in bold, strain represents the MAP-infecting ‘*Ca*. P. solani’ strains.

Nucleotide sequence analyses of *stamp* gene amplicons revealed that ‘*Ca*. P. solani’ strains, identified in MAPs, shared an identical *stamp* gene sequence (GenBank Accession Number PV446481). This sequence was undistinguishable from the sequence variant St5 (GenBank Accession Number FN813256), previously reported in ‘*Ca*. P. solani’ strains infecting several plant hosts, the main insect vector *Hyalesthes obsoletus* Signoret, and additional insect vectors in Europe [31,32,33,47]. Interestingly, ‘*Ca*. P. solani’ strains carrying the *stamp* gene sequence variant St5 were found strictly associated with “bois noir” disease of grapevine in Franciacorta (Brescia province), a viticultural area located close to Farm-1 and Farm-2, examined in this study [33,47]. Phylogenetic analysis showed that St5 ‘*Ca*. P. solani’ strains grouped in the subcluster b-II (Figure 4), including strains associated with the bindweed-related pathosystem [48,49,50]. Considering the polyphagia of ‘*Ca*. P. solani’ insect vectors [43,47], it would be interesting to carry out further studies to investigate if phytoplasma-infected MAP species, identified in this study, could be not only plant hosts but also reservoirs involved in the spread of ‘*Ca*. P. solani’ in agroecosystems.

**Figure 5:**
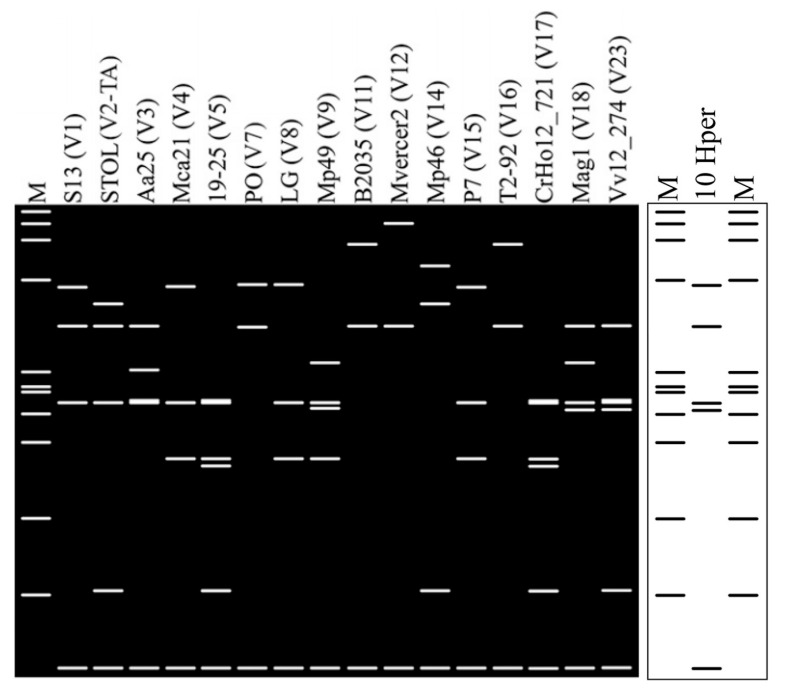
*Rsa*I-RFLP profiles obtained by in silico digestion of *vmp1* gene sequences of ‘*Ca*. P. solani’ strains identified in MAPs and ‘*Ca*. P. solani’ strains representative of previously reported V types.

Nucleotide sequence analyses of *vmp1* gene amplicons revealed that ‘*Ca*. P. solani’ strains, identified in MAPs, shared an identical *vmp1* gene sequence (GenBank Accession Number PV446482). In vitro and in silico digestions showed that all TYPH10F/TYPH10R amplicons obtained in the present study shared a unique *Rsa*I-profile, distinct from those of previously reported V types [28,29] and described as the new V type V24 (Figure 5).

Comparison with previously reported *vmp1* gene sequence variants [30,31,32,33] highlighted that the *vmp1* gene sequence, shared by ‘*Ca*. P. solani’ strains identified in MAPs, have the highest sequence identity values with sequence variants Vm45 (GenBank Accession Number KJ145360) (95.2%), Vm83 (GenBank Accession Number MF182863) (95.3%), Vm87 (GenBank Accession Number MF182867) (95.1%), and Vm44 (GenBank Accession Number HM008612) (95.0%) (Table 2), previously reported in ‘*Ca*. P. solani’ strains infecting grapevine, bindweed, and *H. obsoletus* in Italy [30,31,32,33]. The new *vmp1* gene sequence variant identified in this study was named Vm93.

**Figure 6:**
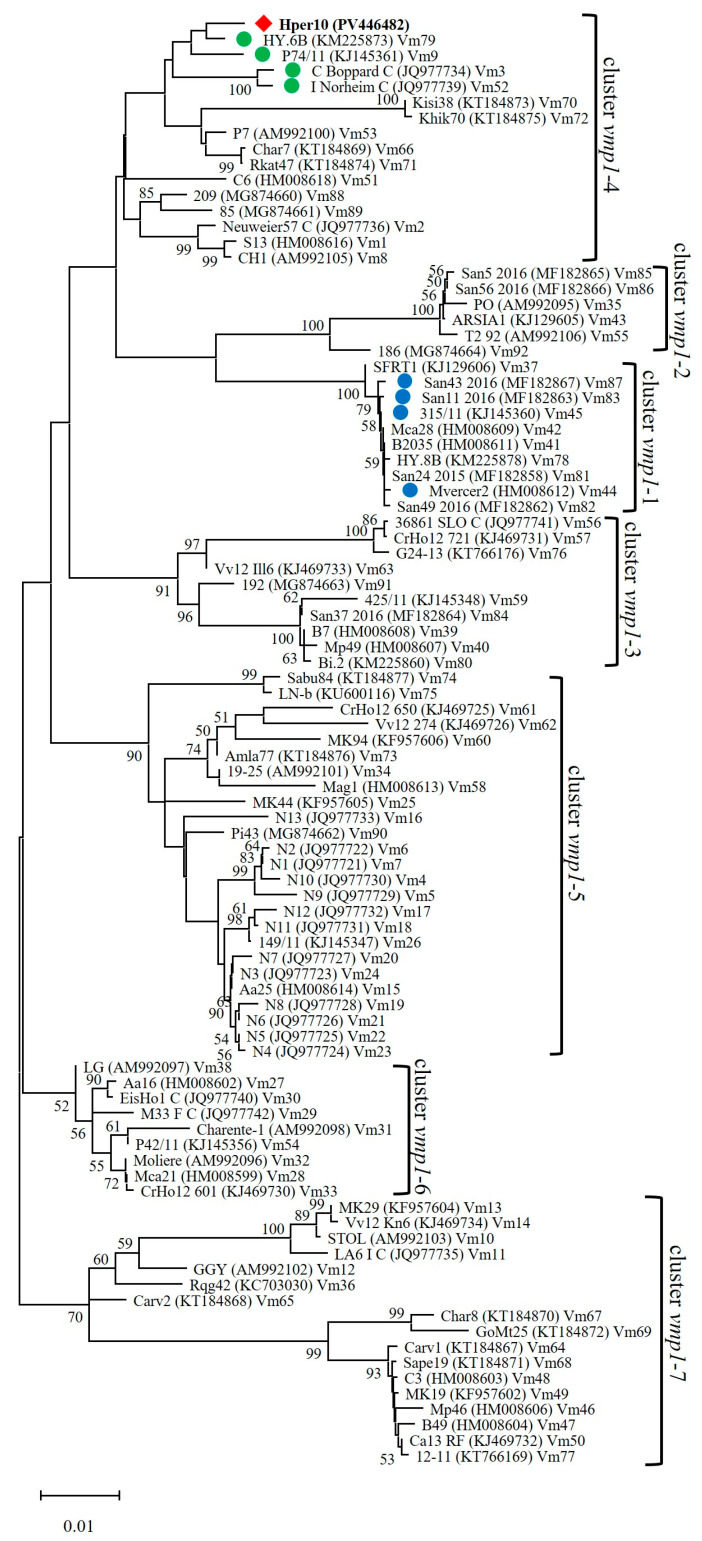
Phylogenetic tree of the *vmp1* gene nucleotide sequence inferred using the minimum evolution method with 1000 bootstrap replicates. The evolutionary distances were computed using the Jukes–Cantor method. The ME tree was searched using the close-neighbor-interchange (CNI) algorithm at a search level of 1. The neighbor-joining algorithm was used to generate the initial tree. This analysis involved 93 nucleotide sequences. There were a total of 1576 positions in the final dataset. The Hper10 (PV446481) strain, written in bold, represents the MAP-infecting ‘*Ca*. P. solani’ strains. Green circles: ‘*Ca*. P. solani’ strains showing best sequence identity with Vm93 without considering gaps; blue circles: ‘*Ca*. P. solani’ strains showing best sequence identity with Vm93 considering gaps.

Phylogenetic analysis showed that Vm93 ‘*Ca*. P. solani’ strains did not group within cluster *vmp1*-1, as expected, along with closest related strains carrying *vmp1* sequence variants Vm45, Vm87, Vm83, and Vm44, but they grouped within cluster *vmp1*-4 along with strains carrying the sequence variants Vm3 (GenBank Accession Number JQ977734) (sequence identity 82.8%), Vm9 (GenBank Accession Number KJ145361) (83.0%), Vm52 (GenBank Accession Number JQ977739) (82.5%), and Vm79 (GenBank Accession Number KM225873) (83.4%) (Table 2) (Figure 6), previously reported in ‘*Ca*. P. solani’ strains infecting grapevine, bindweed, and *H. obsoletus* in Italy, Germany, and France [30,31,32,33]. Such difference between nucleotide sequence identities and phylogenetic clustering is due to the presence of several gaps (insertions/deletions) among the *vmp1* gene sequence of CaPsol strains. In detail, gaps are considered to calculate the Sequence Identity Matrix in BioEdit, while are not considered to generate phylogenetic trees in MEGA X [25,26]. Alignment of *vmp1* nucleotide sequence variant Vm93 with variants showing highest sequence identity (Vm44, Vm45, Vm83, Vm87) and grouping in the same phylogenetic cluster (Vm3, Vm9, Vm52, Vm79) highlighted the presence of three insertions/deletions (ins/del): (i) ins/del-1 in nucleotide position 832–837 (six nucleotides present in variants Vm44, Vm45, Vm83, Vm87); (ii) ins/del-2 in nucleotide position 861-863 (three nucleotides present in variants Vm44, Vm45, Vm83, Vm87); and (iii) ins/del-3 in nucleotide position 958-1206 (248 nucleotides present in variants Vm44, Vm45, Vm83, Vm87, and Vm93). Due to these gaps (ins/del-1 to -3), the length of the *vmp1* nucleotide sequence is 1294 nt in the variants Vm3, Vm9, Vm52, and Vm79; 1542 nt in the variant Vm93; and 1551 nt in the variants Vm44, Vm45, Vm83, and Vm87. If the *vmp1* gene sequence identity is calculated excluding the gaps, considering 1294 nucleotide positions, the variant Vm93 showed the highest identity with variants Vm3, Vm9, Vm52, and Vm79 (average identity 98.7%), carrying an average identity of 94.9% with variants Vm44, Vm45, Vm83, and Vm87 (Table 2), explaining the phylogenetic clustering (Figure 6).

Considering the collective results obtained by *stamp* and *vmp1* gene sequence analyses, it is possible to conclude that a unique, previously unreported ‘*Ca*. P. solani’ genotype (St5/Vm93) has been identified in MAPs from northern Italy.

### 3.4. Vmp1 Gene Sequence Analyses Suggested Recombination Events Among ‘Ca. Phytoplasma solani’ Strains Identified in MAPs

**Figure 7:**
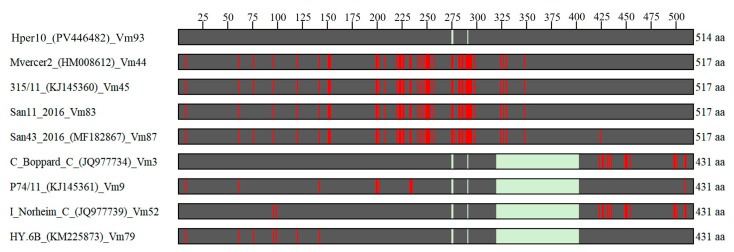
Schematic representation of amino acidic changes (red) and gaps (light green) in Vmp1 protein sequences of ‘*Ca*. P. solani’ strains carrying the variant Vm93 (identified in this study) and closest variants.

Analyses of the nucleotide *vmp1* gene and in silico-translated amino acidic Vmp1 protein sequences (Figure 7) revealed that, considering both SNPs and gaps (ins/del), sequence variant Vm93, shared by ‘*Ca*. P. solani’ strains identified in MAPs in this study, is more similar to variants Vm3, Vm9, Vm52, and Vm79 in the first part of the sequence (nucleotides 1–957, amino acids 1–319), while is more similar to variants Vm44, Vm45, Vm83, and Vm87 in the second part of the sequence (nucleotides 958–1551, amino acids 320–517) (Table 3). As described above, this difference is due mainly to the ins/del-3 region (nucleotides 958-1206, amino acids 320–402) present in variant Vm93 and variants Vm44, Vm45, Vm83, and Vm87.

**Figure 8:**
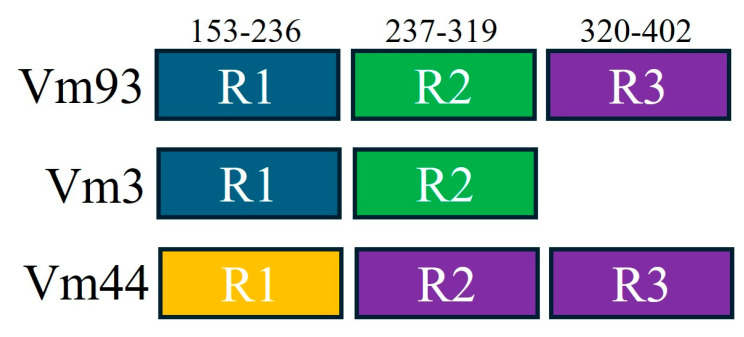
Repeated domains within Vmp1 protein sequences of ‘*Ca*. P. solani’ strains carrying the variant Vm93 (identified in this study) and closest variants Vm3 and Vm44. Colors are identical in repeated domains sharing sequence identity higher than 90%.

As reported in a previous study [28], Vmp1 sequence analysis identified three repeated domains, here named R1 (amino acids 152-236), R2 (amino acids 237–319), and R3 (amino acids 320-402). Domains R1 and R2 were identified in Vm93 and in the other closest variants, while R3 was present only in Vm93 and variants Vm44, Vm45, Vm83, and Vm87 (Figure 8).

Comparing amino acidic sequences of R1 to R3 domains of sequence variants Vm93, Vm3 (representative of variants Vm3, Vm9, Vm52, and Vm79) and Vm44 (representative of variants Vm44, Vm45, Vm83, and Vm87), it was found that (i) domain R1 is identical in variant Vm93 and Vm3 (identity of 84.5% vs. Vm44); (ii) domain R2 is identical in variant Vm93 and Vm3 (identity of 72.2% vs. Vm44); (iii) domain R1 and R2 in variants Vm93/Vm3 and in variant Vm44 have an identity of 69.0% and 79.7%, respectively; (iv) domain R3 (absent in Vm3) in variants Vm93 and Vm44 has an identity of 95.1%; and (v) domain R2 of Vm44 has an identity of 98.7% and 93.9% with domain R3 of variants Vm44 and Vm93, respectively (Figure 8, Table 4).

Interestingly, previous studies showed that (i) the *vmp1* gene of ‘*Ca*. P. solani’ has low homology with a *Mycoplasma agalactiae* surface-variable lipoprotein gene, with its variability determined by DNA rearrangements [51,52]; (ii) the *vmp1* gene of ‘*Ca*. P. solani’, such as homologous *vmpA* and *vmpB* genes of Flavescence dorée phytoplasma [53], is under positive selection and has repeated domains, as in Scarps (*Spiroplama citri* adhesion-related proteins) genes [54]; (iii) domain duplication and deletion can occur by RecA-dependent recombination mechanisms [55,56]; (iv) the *recA* gene, encoding RecA protein involved in DNA repair mechanism and recombination, is present in the ‘*Ca*. P. solani’ genome [57]; and (v) differences in surface protein repeated domains can be related to duplication and divergent evolution [58], as hypothesized for VmpA and VmpB of Flavescence dorée phytoplasma [53].

Based on evidence obtained in this and previous studies, it is reasonable to hypothesize that, in ‘*Ca*. P. solani’ strains identified in MAPs (Vm93) and in other *vmp1* sequence variants analyzed in this study, *vmp1* repeated domains R1 and R2 were generated by relatively ancient duplication and divergent evolution, as suggested in other phytoplasma [53]. Considering the sequence identity between Vmp1 domains R2 and R3 in ‘*Ca*. P. solani’ strains carrying the variants Vm93 and Vm44 (representative of other variants Vm45, Vm83, and Vm87) (Figure 8, Table 4), it is probable that (i) domain R3 in Vm44 (Vm45, Vm83, and Vm87) was generated by relatively recent duplication of domain R2 (identity 98.7%) and (ii) domain R3 in Vm93 was acquired by recombination with Vm44, Vm45, Vm83, or Vm87 (identity 95.1%). Recombination is supported by the presence of the *recA* gene in ‘*Ca*. P. solani’ genome, and by the common epidemiological bindweed-related pathosystem shared by Vm93 and Vm44 ‘*Ca*. P. solani’ strains.

Considering that (i) variations in repeated domains of *vmpA* and *vmpB* genes determined differences in transmissibility of Flavescence dorée phytoplasma strains by vectors [53], and (ii) several insect vectors of ‘*Ca*. P. solani’ were recently reported in northern Italy [47], it will be intriguing to investigate if ‘*Ca*. P. solani’ genotype St5/Vm93 can be involved in epidemiological pattern(s) related to specific insect vector(s), and to determine if symptomless phytoplasma-infected MAPs can constitute reservoir plants (inoculum sources) for ‘*Ca*. P. solani’ epidemics in other crops. Moreover, as phytoplasmas are known to modify the secondary metabolites in MAPs [8,9], it should be interesting to evaluate the metabolite content in symptomatic and symptomless MAPs infected by ‘*Ca*. P. solani’.

## Figures and Tables

**Table 1:** Collected and phytoplasma-infected medicinal and aromatic plants.

Farm—Location	Species	Family	No. of Plants	
			Collected	PCR Positive
1—Rovagnate (LC)	*Achillea millefolium* L.	Asteraceae	1	0
	*Artemisia absinthium* L.	Asteraceae	4	4
	*Calendula officinalis* L.	Asteraceae	5	0
	*Filipendula ulmaria* (L.) Maxim.	Rosaceae	5	4
	*Hypericum perforatum* L.	Hypericaceae	7	7
	*Inula salicina* L.	Asteraceae	5	0
	*Lavandula* sp. L.	Lamiaceae	4	3
	*Matricaria recutita* L.	Asteraceae	3	3
	*Melissa officinalis* L.	Lamiaceae	4	4
	*Mentha* sp. L.	Lamiaceae	4	3
	*Rosmarinus officinalis* L.	Lamiaceae	4	4
	*Salvia officinalis* L.	Lamiaceae	2	2
	*Urtica dioica* L.	Urticaceae	6	1
	*Valeriana officinalis* L.	Caprifoliaceae	6	5
		Farm-1 total	60	40
2—Solto Collina (BG)	*Echinacea purpurea* (L.) Moench	Asteraceae	5	0
	*Hypericum perforatum* L.	Hypericaceae	5	5
	*Lavandula* sp. L.	Lamiaceae	7	6
	*Levisticum officinale* W.D.J.Koch	Apiaceae	2	2
	*Melissa officinalis* L.	Lamiaceae	3	3
	*Mentha* sp. L.	Lamiaceae	5	5
	*Monarda didyma* L.	Lamiaceae	5	1
	*Nepeta cataria* L.	Lamiaceae	15	7
	*Urtica dioica* L.	Urticaceae	6	0
		Farm-2 total	53	29
		Overall total	113	69

**Table 2:** Sequence identity and mutations among *vmp1* gene sequence variants.

*vmp1* Gene Variant	No. SNPs (Sequence Identity) vs. *vmp1* Sequence Variant Vm93 (1542 nt)	
	with Gaps (Insertion/Deletion) (1551 nt)	Without Gaps (Insertion/Deletion) (1294 nt)
Vm44 (1551 nt)	77 (95.0%)	68 (94.7%)
Vm45 (1551 nt)	74 (95.2%)	65 (95.0%)
Vm83 (1551 nt)	73 (95.3%)	64 (95.1%)
Vm87 (1551 nt)	75 (95.1%)	66 (94.9%)
Vm3 (1294 nt)	266 (82.8%)	18 (98.6%)
Vm9 (1294 nt)	263 (83.0%)	15 (98.8%)
Vm52 (1294 nt)	271 (82.5%)	23 (98.2%)
Vm79 (1294 nt)	257 (83.4%)	9 (99.3%)

**Table 3:** Number of single nucleotide polymorphisms and amino acidic changes in two regions of Vmp1 protein sequences of ‘*Ca*. P. solani’ carrying the sequence variant Vm93 and closest variants.

*vmp1* Gene Variant	Differences vs. *vmp1*/Vmp1 Sequence Variant Vm93			
	Nt 1-957	Aa 1-319	Nt 958-1551	Aa 320-517
	No. SNPs *	No. aa-Change *	No. SNPs *	No. aa-Change *
Vm44	71 (9)	44 (3)	6 (0)	4 (0)
Vm45	68 (9)	44 (3)	6 (0)	4 (0)
Vm83	67 (9)	43 (3)	6 (0)	4 (0)
Vm87	68 (9)	45 (3)	7 (0)	5 (0)
Vm3	0 (0)	0 (0)	266 (248)	98 (83)
Vm9	14 (0)	9 (0)	249 (248)	84 (83)
Vm52	5 (0)	2 (0)	266 (248)	98 (83)
Vm79	9 (0)	7 (0)	248 (248)	83 (83)

* Number of gaps (insertion/deletion) indicated between brackets.

**Table 4:** Sequence identity among repeated domains in Vmp1 proteins of ‘*Ca*. P. solani’ carrying the sequence variant Vm93 and closest variants.

#	Vmp1 Domain	1	2	3	4	5	6	7	8
1	Vm93_R1_aa153-236	ID							
2	Vm3_R1_aa153-236	1	ID						
3	Vm44_R1_aa153-236	0.845	0.845	ID					
4	Vm93_R2_aa237-319	0.69	0.69	0.738	ID				
5	Vm3_R2_aa237-319	0.69	0.69	0.738	1	ID			
6	Vm44_R2_aa237-319	0.666	0.666	0.797	0.722	0.722	ID		
7	Vm93_R3_aa320-402	0.702	0.702	0.821	0.746	0.746	0.939	ID	
8	Vm44_R3_aa320-402	0.666	0.666	0.785	0.722	0.722	0.987	0.951	ID

## Data Availability

The original contributions presented in this study are included in the article. Further inquiries can be directed at the corresponding author.
